# Eph/Ephrin Signaling Controls Progenitor Identities In The Ventral Spinal Cord

**DOI:** 10.1186/s13064-017-0087-0

**Published:** 2017-06-08

**Authors:** Julien Laussu, Christophe Audouard, Anthony Kischel, Poincyane Assis-Nascimento, Nathalie Escalas, Daniel J. Liebl, Cathy Soula, Alice Davy

**Affiliations:** 1 Centre de Biologie du Développement (CBD), Centre de Biologie Intégrative (CBI), Université de Toulouse, CNRS, UPS, 118 Route de Narbonne, 31062 Toulouse, France; 2 Present address: CRBM, 1919 route de Mende, 34293 Montpellier, France; 3 University of Miami Miller School of Medicine, The Miami Project to Cure Paralysis, 1095 NW 14th Terrace, Miami, FL R-48 USA

**Keywords:** Ephrins, Neural tube, Progenitors, Fate, Motor neurons, Sonic hedgehog, Mouse

## Abstract

**Background:**

In the vertebrate spinal cord, motor neurons (MN) are generated in stereotypical numbers from a pool of dedicated progenitors (pMN) whose number depends on signals that control their specification but also their proliferation and differentiation rates. Although the initial steps of pMN specification have been extensively studied, how pMN numbers are regulated over time is less well characterized.

**Results:**

Here, we show that ephrinB2 and ephrinB3 are differentially expressed in progenitor domains in the ventral spinal cord with several Eph receptors more broadly expressed. Genetic loss-of-function analyses show that ephrinB2 and ephrinB3 inversely control pMN numbers and that these changes in progenitor numbers correlate with changes in motor neuron numbers. Detailed phenotypic analyses by immunostaining and genetic interaction studies between ephrinB2 and Shh indicate that changes in pMN numbers in ephrin mutants are due to alteration in progenitor identity at late stages of development.

**Conclusions:**

Altogether our data reveal that Eph:ephrin signaling is required to control progenitor identities in the ventral spinal cord.

**Electronic supplementary material:**

The online version of this article (doi:10.1186/s13064-017-0087-0) contains supplementary material, which is available to authorized users.

## Background

In vertebrates, motor neurons (MN) innervating skeletal muscles are born in the ventral neural tube, the future spinal cord, from a pool of progenitors located in the ventricular zone. As for other neuronal subtypes, the production of stereotyped numbers of MN requires the integration of different processes such as specification and proliferation of progenitors, followed by cell cycle exit and differentiation [1]. While these processes are common to the genesis of all neuronal subtypes throughout the central nervous system, one distinguishing feature of MN development is the fact that progenitor specification is dependent on their spatial organization within the neural tube. Indeed, the vertebrate neural tube is organized along its dorsoventral axis in different progenitor domains which first give rise to distinct neuronal subtypes and later on to subtypes of glial cells. Combinatorial positional information provided by graded Sonic Hedgehog (Shh), Wnt, BMP and FGF signaling induces the regionalized expression of homeodomain and helix-loop-helix identity transcription factors (iTFs) in different progenitor domains [1]. For instance, progenitors of motor neurons (pMNs) express the iTF Olig2 while adjacent progenitors (p3) which give rise to v3 interneurons express the iTF Nkx2.2. Ventral patterning of the spinal cord, including specification of pMN and p3 progenitors, is controlled by the morphogen Sonic Hedgehog (Shh) produced by the notochord and the floor plate [2]. In addition to doses, different exposure times to Shh modulates the expression of these iTFs in progenitors, thus specifying the distinct progenitor identities [3, 4]. Specifically, the progressive emergence of a gene regulatory network (GRN) composed of three transcription factors- Pax6, Olig2 and Nkx2.2 whose expression is refined by cross-repressive interactions interprets graded Shh signaling, to control the size of the p3 and pMN progenitor domains [5, 6]. Although early steps of progenitor specification are fairly well characterized, mechanisms that ensure stereotypy in the size of progenitor domains as the tissue grows are less well understood. These mechanisms include maintenance of progenitor identities [4, 7, 8] as well as control of proliferation and differentiation rates that vary between progenitor types and over time [9]. In addition, mechanisms such as cell sorting have been shown to participate in defining and/or maintaining domain boundaries thus indirectly contributing to pattern progenitor domains [10–13].

Eph:ephrin signaling is a cell-to-cell communication pathway that has been implicated in numerous developmental processes [14, 15]. A distinctive feature of Eph:ephrin signaling is its ability to trigger forward signaling downstream of Eph receptors and reverse signaling downstream of ephrins. One of its major biological functions is to control cell adhesion and repulsion events in developing and adult tissues thus leading to the establishment and/or maintenance of axon tracts, tissue organization and patterning [16, 17]. In addition, Eph:ephrin signaling has been shown to control various aspects of neural progenitor development and homeostasis in the developing and adult mammalian cortex including self-renewal, proliferation, quiescence and differentiation [18]. In the developing spinal cord, the role of Eph:ephrin signaling has been prominently studied in post-mitotic neurons, specifically in axon guidance and fasciculation of MNs [19–21], as a consequence, virtually nothing is known on the function of this pathway in spinal progenitors. Here, we show that two B-class ephrins, ephrinB2 and ephrinB3 are differentially expressed in pMN and p3 progenitors. Loss of function analyses indicate that expression of ephrinB2 and ephrinB3 is not required for initial specification of these progenitors. However, at later developmental stages, expression of ephrinB2 and ephrinB3 is essential to maintain appropriate numbers of pMN and p3 progenitors. Interestingly, ephrinB2 and ephrinB3 mutants exhibit opposite phenotypes, matching their opposite differential expression patterns. Detailed analyses of ephinB2 mutants indicate that the change in pMN number is not due to a change in proliferation or differentiation rates. Rather, our data shows that *Efnb2* interacts with *Shh* to control the ratio between pMN and p3 progenitors. Lastly, loss of ephrinB3 -but not ephrinB2- leads to pMN and p3 progenitor intermingling. Altogether our data suggests that Eph:ephrin signaling plays a role in controlling progenitor identity.

## Methods

### Mice

Ephrin mutant mice were maintained in a mixed background and genotyped by PCR. The mouse lines *Shh* ^*ko*^, *Efnb3* ^*ko*^, *Efnb2* ^*lox*^ and *Efnb2* ^*GFP*^ have been described previously [22–24]. The *Olig2-Cre* mouse line [3] was maintained in a pure C57Bl6/J genetic background. For *Efnb2* cKO*,* control genotypes used in the study include *Efnb2* ^*lox/lox*^, *Efnb2* ^*lox/GFP*^, *Efnb2* ^*+/GFP*^ and *Efnb2* ^*+/GFP*^ *; Olig2-Cre*. For *Efnb3* KO*,* control genotypes are always *Efnb3* ^*+/-*^. E0.5 is defined as the day on which a vaginal plug was detected.

### In Situ Hybridization

In situ hybridization was performed using standard protocols on 70μm vibratome sections at brachial level. Antisense RNA probes labeled with digoxigenin were used to detect in vivo gene expression with a 72 h incubation time.

### Immunostaining

All analyses for *Efnb2* cKO were performed on control and mutant littermates collected from at least two different litters. On the other hand, control and *Efnb3* mutant embryos were collected from independent litters. The number of embryo analyzed for each immunostaining and each developmental stage is indicated in the figure legends. To avoid bias in rostro-caudal axis, data was collected on thick vibratome sections covering the entire brachial region (600 μm). Antibody staining was performed following standard protocol on 70μm vibratome sections of mouse embryos at brachial level. For BrdU incorporation, pregnant dams were injected with BrdU (10mg/ml; 100mg/kg) with intraperitoneal injection. After 1 h, embryos were dissected in cold PBS and processed for subsequent immunostaining.

Antibodies used were: goat anti-Nkx2.2 (1/100, Santa Cruz Biotechnology); rabbit anti-Olig2 (1/1000, Sigma); mouse anti-Islet1/2, 39-4D5 (1/50, DSHB); rabbit anti-Foxp1 (1/200, Abcam), rabbit anti-P-H3 (1/1000, Millipore), rabbit anti-EphA4 (1/100, Santa Cruz Biotechnology), goat anti-EphB2 (1/50, R&D Systems), Tuj1 (1/1000, Covance). All secondary antibodies were from Jackson ImmunoResearch (1/1000).

### Image processing and quantification

Images were collected on a Leica SP5 confocal microscope or Nikon eclipse 80i microscope for *in situ* hybridization data. Cell numbers were collected blindly on 5 vibratome sections (*n*=25 confocal Z-sections) per embryo and at least 2000 nuclei were recorded per embryo. The number of embryo analyzed for each immunostaining and each developmental stage is indicated in the figure legends. Acquisitions of nuclei 2D positions and semi quantitative analyses of fluorescence intensity were performed using Fiji [25]. Spatial distribution of progenitor subtypes was quantified using the R Project (http://www.r-project.org/), see Additional file 1: (Sup Code) for details on the code.

### Statistical Analysis

For all analyses sample size was estimated empirically. Sample sizes are indicated in Figure legends and further details are provided in Additional file 2: Table S1. Statistical analyses were performed with GraphPad, using Mann-Whitney-Wilcoxon test or ANOVA, depending on the data set. *P*<0.05 was considered statistically significant.

## Results

### EphrinB2 and ephrinB3 exhibit restricted expression in progenitors of the ventral spinal cord.

**Fig. 1.**
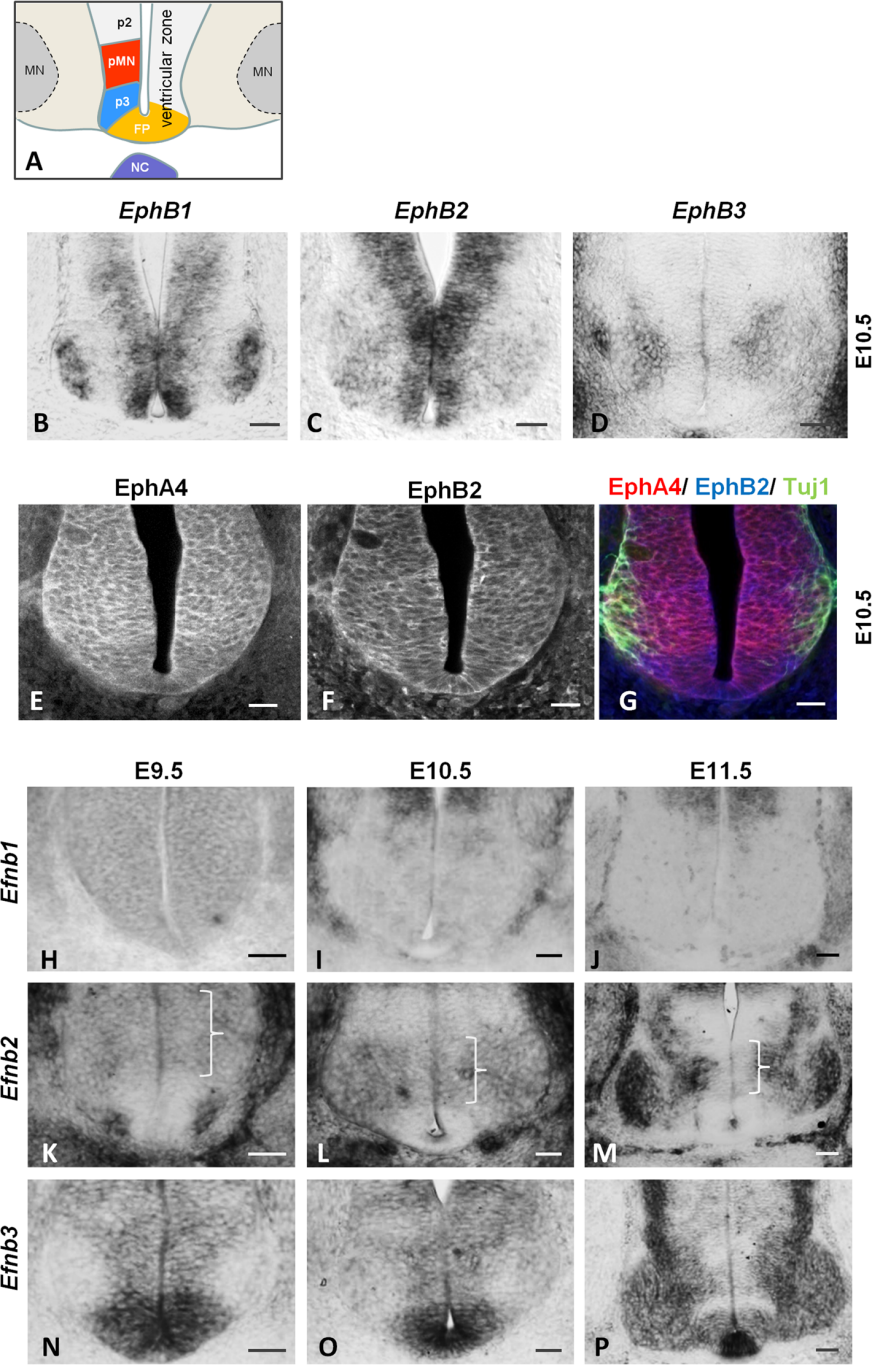
Eph receptors and ephrins are expressed in progenitors of the ventral spinal cord. **a**. Schematic representation of the ventral spinal cord at E10.5. Progenitors are located in the ventricular zone, three progenitor domains are shown: p2, pMN and p3. Differentiated motor neurons (MN) are located laterally in the mantle zone. **b-d**. Expression of *EphB1* (**b**), *EphB2* (**c**) and *EphB3* (**d**) was monitored by in situ hybridization on transverse sections of E10.5 embryos. Scale bars: 50 μm. **e-g**. Transverse sections of E10.5 embryos were immunostained to detect EphA4 ((**e**), *red*), EphB2 ((**f**)*, blue*) and differentiated neurons (Tuj1, green in (**g**)). Scale bars: 40 μm. (**h-p**). Expression of *Efnb1* (**h-j**), *Efnb2* (**k-m**) and *Efnb3* (**n-p**) was monitored by in situ hybridization on transverse sections of E9.5, E10.5 and E11.5 embryos, as indicated. Scale bars: 50 μm. Brackets indicate domains of *Efnb2* expression in progenitors. FP: floor plate, NC: notochord.

**Fig. 2.**
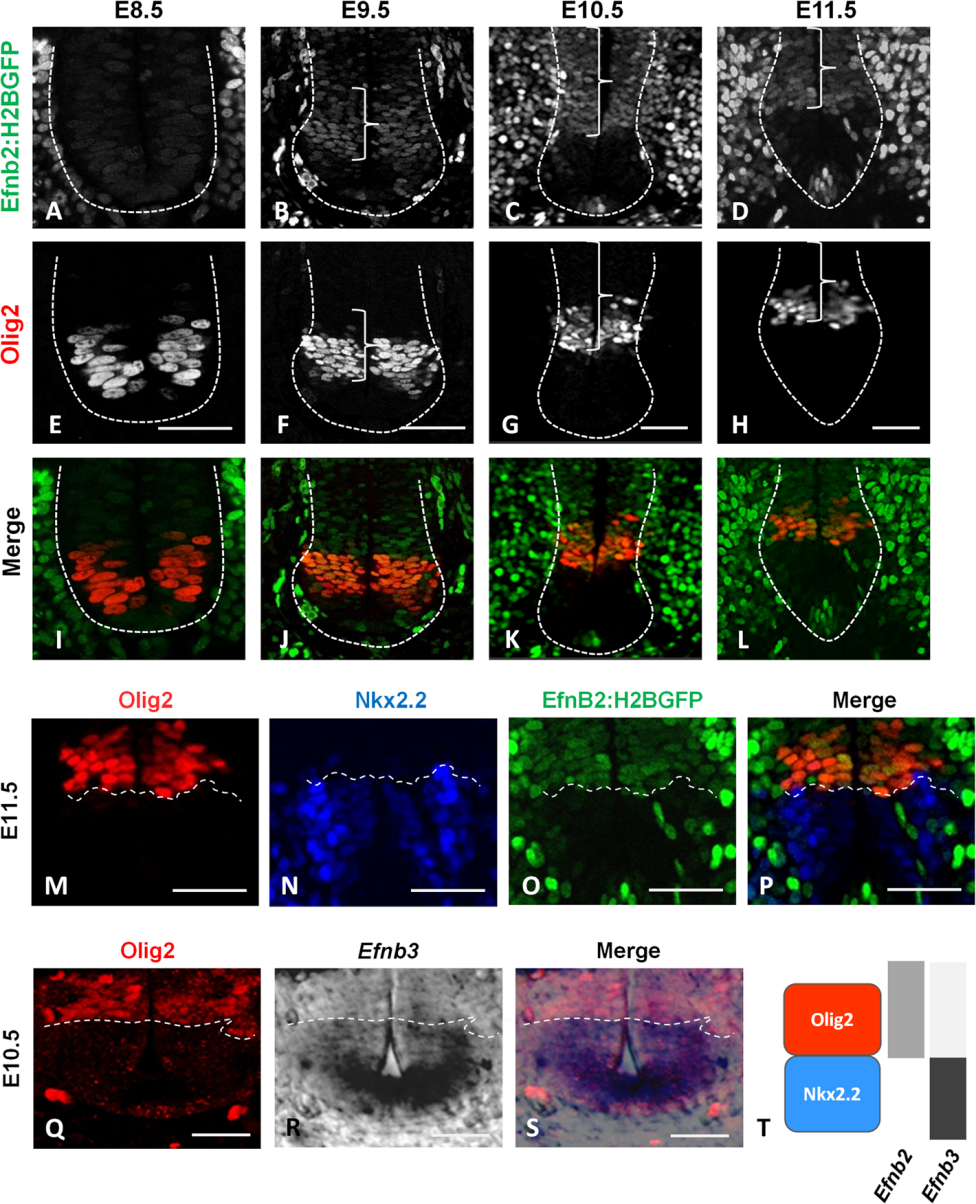
EphrinB2 and ephrinB3 are expressed in complementary domains in progenitors of the ventral spinal cord. **a-l**. Transverse sections of *Efnb2* ^*+/GFP*^ embryos at E8.5, E9.5, E10.5 and E11.5 (as indicated) were immunostained to detect Olig2 (**e-h**). Epifluorescence is shown on (**a-d**) and merged images are shown on (**i-l**). Scale bars: 50 μm. Dashed lines highlight the ventricular zone and brackets indicate domains of *Efnb2:H2BGFP* expression in progenitors. **m-p**. Transverse sections of *Efnb2* ^*+/GFP*^ E11.5 embryos were immunostained to detect Olig2 (**m**) and Nkx2.2 (**n**). Epifluorescence is shown in (**o**) and a merged image is shown in (**p**). The dashed line marks the p3/pMN boundary. Scale bars: 50 μm. **q-s**. Transverse sections of wild type E10.5 embryos were processed for Olig2 immunostaining (**q**) and for *Efnb3* in situ hybridization (**r**). A merged image is shown in (**s**). The dashed line marks the p3/pMN boundary. Scale bars: 25 μm. T. Schematic representation of *Efnb2* and *Efnb3* expression in relation to pMN and p3 progenitors domains at E11.5.

A survey of members of the B-type Eph receptor family in the mouse ventral spinal cord (Fig. 1a) indicated that spinal progenitors co-express several EphB receptors, as well as EphA4, as shown by in situ hybridization (Fig. 1b-d) and immunofluorescence (Fig. 1e-g). Concerning B-type ephrin ligands, in situ hybridization at different developmental stages reveals that while *Efnb1* is not expressed at significant levels in progenitors of the ventral spinal cord (Fig. 1h-j), both *Efnb2* and *Efnb3* are expressed in subsets of these cells. More precisely, at all stages analyzed, *Efnb2* is expressed by progenitors located at an intermediate dorso-ventral position within the spinal cord, its expression never extending to the ventral-most region (Fig. 1k-m). Conversely, expression of *Efnb3* is highest in the ventral-most region of the spinal cord at all stages analyzed, with a lower expression extending more dorsally (Fig. 1n-p). Because *Efnb2* and *Efnb3* were expressed in distinct progenitor domains of the spinal cord, we asked whether these corresponded to progenitors with distinct identities, namely pMN progenitors expressing Olig2 and p3 progenitors expressing Nkx2.2. Since the expression of *Efnb2* in progenitors of the ventral neural tube detected by in situ hybridization was low, we took advantage of a reporter mouse line that expresses H2BGFP under the control of the *Efnb2* endogenous promoter [22]. The benefit of this reporter strategy is that H2BGFP accumulates in the nucleus thus highlighting low domains of expression and facilitating co-expression analyses. In accordance with in situ hybridization data, H2BGFP expression was detected in a restricted population of neural progenitors from E9.5 to E11.5 (Fig. 2a-d). Co-staining with Olig2 showed that the expression domain of *Efnb2* overlapped with the Olig2^+^ (pMN) domain (Fig. 2a-l). Co-staining with Olig2 and Nkx2.2, the iTFs for pMN and p3 respectively, showed that the ventral boundary of *Efnb2* expression strictly corresponds to the p3/pMN boundary (Fig. 2m-p). Conversely, in situ hybridization for *Efnb3* followed by Olig2 immunostaining showed that the highest domain of *Efnb3* expression corresponds to Olig2^-^ floor plate and p3 progenitors (Fig. 2q-s). Altogether, these expression analyses indicate that all progenitors of the ventral spinal cord co-express several Eph receptors and reveal that ephrinB2 and ephrinB3 are differentially expressed in pMN and p3 progenitors (Fig. 2t).

### EphrinB2 controls the number of pMN and their progeny

**Fig. 3.**
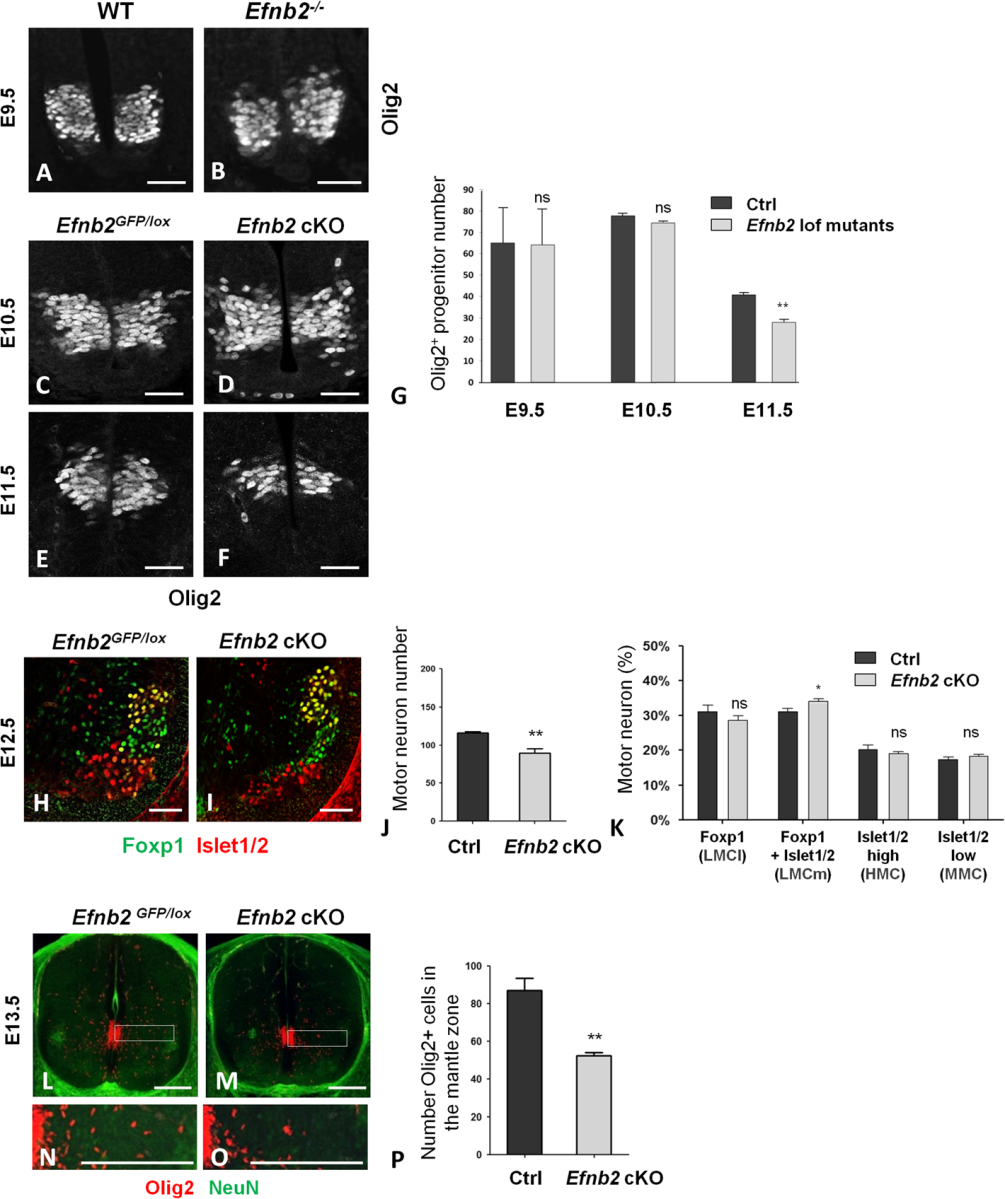
EphrinB2 controls the number of pMN progenitors and their progeny. **a**, **b**. Transverse sections of wild type (**a**) and *Efnb2* ^*-/-*^ (**b**) E9.5 embryos were immunostained to detect Olig2. **c**, **d**. Transverse sections of *Efnb2* ^*lox/GFP*^ (**c**) and *Efnb2* ^*lox/GFP*^ *;Olig2-Cre* (**d**) E10.5 embryos were immunostained to detect Olig2. **e**, **f**. Transverse sections of *Efnb2* ^*lox/GFP*^ (**e**) and *Efnb2* ^*lox/GFP*^ *;Olig2-Cre* (**f**) E11.5 embryos were immunostained to detect Olig2. **g**. The number of Olig2+ progenitors was quantified for all genotypes. Error bars indicate s.e.m. (*n*=3 embryos per genotype at E9.5; *n*=4 embryos per genotype at E10.5; *n*=5 embryos per genotype at E11.5); **P<0.01, ns= non significant (Mann-Whitney-Wilcoxon test). **h**, **i**. Transverse sections of E12.5 *Efnb2* ^*lox/GFP*^ (**h**) and *Efnb2* ^*lox/GFP*^ *; Olig2-Cre* (**i**) embryos were immunostained to detect Foxp1 (*green*) and Islet 1/2 (*red*). **j**. Quantification of the total number of motor neurons (Foxp1^+^ and Islet 1/2^+^) in both genotypes. **k**. Repartition of motor neurons in motor columns in both genotypes. Error bars indicate s.e.m. (*n*=6 embryos per group); *P<0.05; **P<0.01; ns= non significant (Mann-Whitney-Wilcoxon test). **l**-**o**. Transverse sections of E13.5 *Efnb2* ^*lox/GFP*^ (**l**, **n**) and *Efnb2* ^*lox/GFP*^ *; Olig2-Cre* (**m**, **o**) embryos were immunostained to detect Olig2 (*red*) and NeuN (*green*). **n**, **o** are zoomed areas indicated by a box in **m**, **n** respectively. **p**. Quantification of the total number of Olig2^+^ cells in the mantle zone in both genotypes. Error bars indicate s.e.m. (*n*=4 embryos per group); **P<0.01 (Mann-Whitney-Wilcoxon test). Scale bars A-I: 50 μm; L-O: 200 μm.

Based on the expression of ephrinB2 in pMN but not p3 progenitors we hypothesized that it may be required for pMN development. We thus generated *Efnb2* loss-of-function mutant embryos and quantified the number of Olig2^+^ progenitors at three different developmental stages. Since *Efnb2* ^*-/-*^ (KO) embryos exhibit precocious lethality (E10.5) due to cardiovascular defects [26, 27] to analyze later stages of development we generated *Efnb2* conditional mutant embryos, using the *Olig2-Cre* mouse line (*Efnb2* ^*lox/lox*^ *; Olig2-Cre* thereafter called cKO). At E9.5 and E10.5, no difference was observed in the number of Olig2^+^ progenitors when comparing wild type and *Efnb2* KO or *Efnb2* cKO (Fig. 3a-d, g). On the contrary, at E11.5, the number of Olig2^+^ progenitors was significantly decreased in *Efnb2* cKO (Fig. 3e-g), indicating that ephrinB2 is not required for initial pMN specification but is necessary at later stages to control the number of pMN progenitors. The decrease in the number of Olig2^+^ progenitors between E10.5 and E11.5 which is observed in wild type embryos is partly driven by differentiation of these cells into MN [9], raising the possibility that the decrease in the number of pMN progenitors in absence of ephrinB2 could be due to increased differentiation. We thus assessed the number of pMN progeny in wild type and *Efnb2* mutant embryos. In the spinal cord, pMN first give rise to MN which settle in specific motor columns in the mantle zone and second, after the neuroglial transition, pMN give rise to oligodendrocyte precursors (OLP) that maintain Olig2 expression and migrate in the mantle zone. Immunostaining for Islet1/2 and Foxp1 to label MN showed that the reduction in pMN numbers correlates with a reduction in the total number of MN in E12.5 *Efnb2* cKO (Fig. 3h-j), which is not consistent with increased differentiation. To assess whether one subtype of MN was preferentially affected, we used different combination of Foxp1 and Islet1/2 to discriminate different motor columns. These quantifications showed that the reduction of total MN number does not correlate with reduction of one specific motor column, however, we observed that within this reduced pool of MN, slightly more LMCm MN were present in *Efnb2* cKO (Fig. 3k). Next we assessed the number of OLP by quantifying Olig2^+^ nuclei in the mantle zone of E13.5 *Efnb2* cKO. Similar to what was observed for MN, loss of *Efnb2* in pMN correlates with a decrease in OLP numbers (Fig. 3l-p). Altogether, this data reveal that ephrinB2 is required to produce a stereotyped number of pMN and of their progeny by mechanisms likely independent of differentiation.

### *Efnb2* interacts with *Shh* to control the ratio between pMN and p3 progenitors

**Fig. 4.**
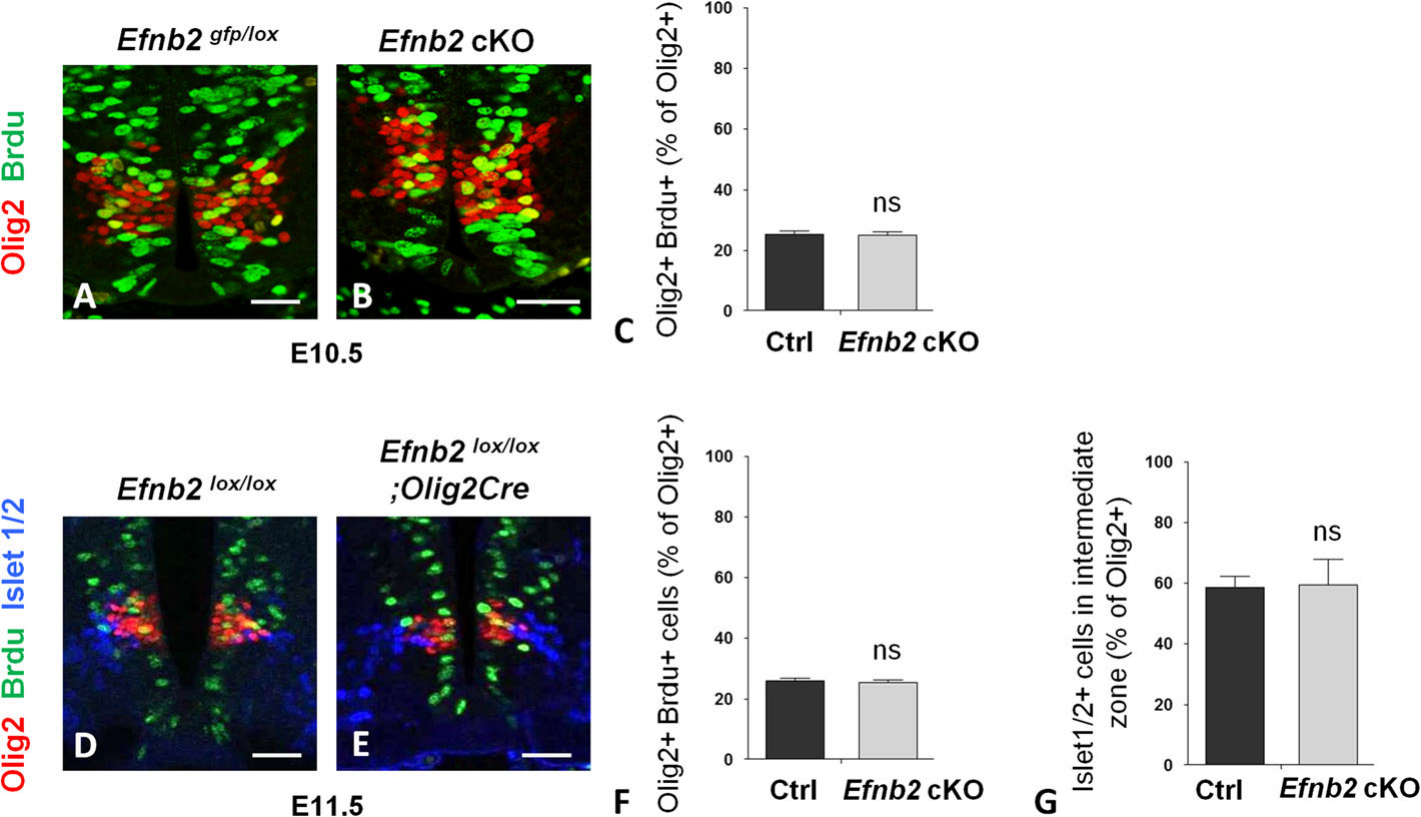
No change in pMN proliferation or differentiation rates in absence of ephrinB2. **a**, **b**. Transverse sections of E10.5 *Efnb2* ^*lox/GFP*^ (**a**) and *Efnb2* ^*lox/GFP*^ *; Olig2-Cre* (**b**) embryos were immunostained to detect Olig2 (*red*) and BrdU (*green*). **c**. Quantification of BrdU^+^ nuclei in the Olig2^+^ population in both genotypes. **d**, **e**. Transverse sections of E11.5 *Efnb2* ^*lox/GFP*^ (**d**) and *Efnb2* ^*lox/GFP*^ *; Olig2-Cre* (**e**) embryos were immunostained to detect Olig2 (*red*), Islet 1/2 (blue) and BrdU (*green*). **f**. Quantification of BrdU^+^ nuclei in the Olig2^+^ population in both genotypes. **g**. Quantification of Islet 1/2^+^ nuclei in the intermediate zone relative to the Olig2^+^ population in both genotypes. Error bars indicate s.e.m. (*n*=4 embryos per group); ns= non significant (Mann-Whitney-Wilcoxon test). Scale bars: 50 μm.

To characterize the underlying causes of the decreased pMN number in ephrinB2 mutants, we first tested whether this reduction was due to altered rates of proliferation of pMN. We performed BrdU incorporation and immunostaining for Olig2 and quantified BrdU^+^/Olig2^+^ progenitors at two different developmental stages to monitor proliferation. At both stages, no difference in pMN proliferation was observed in *Efnb2* cKO compared to control embryos (Fig. 4a-f). We also assessed apoptosis and observed no change in the number of cleaved caspase positive pMN at E10.5 and E11.5 (data not shown). In addition, to confirm that the rate of pMN differentiation was unchanged in *Efnb2* cKO, we performed co-immunostaining for Olig2 and for the motor neuron (MN) marker Islet1/2 and quantified the fraction of Islet1/2^+^ nuclei at the basal side of the ventricular zone (intermediate zone) which represent newborn MN. As expected, no difference was observed between control and *Efnb2* cKO embryos (Fig. 4d, e, g). These results show that the reduction in pMN number in absence of ephrinB2 is not due to alteration in their rate of proliferation or differentiation.

**Fig. 5.**
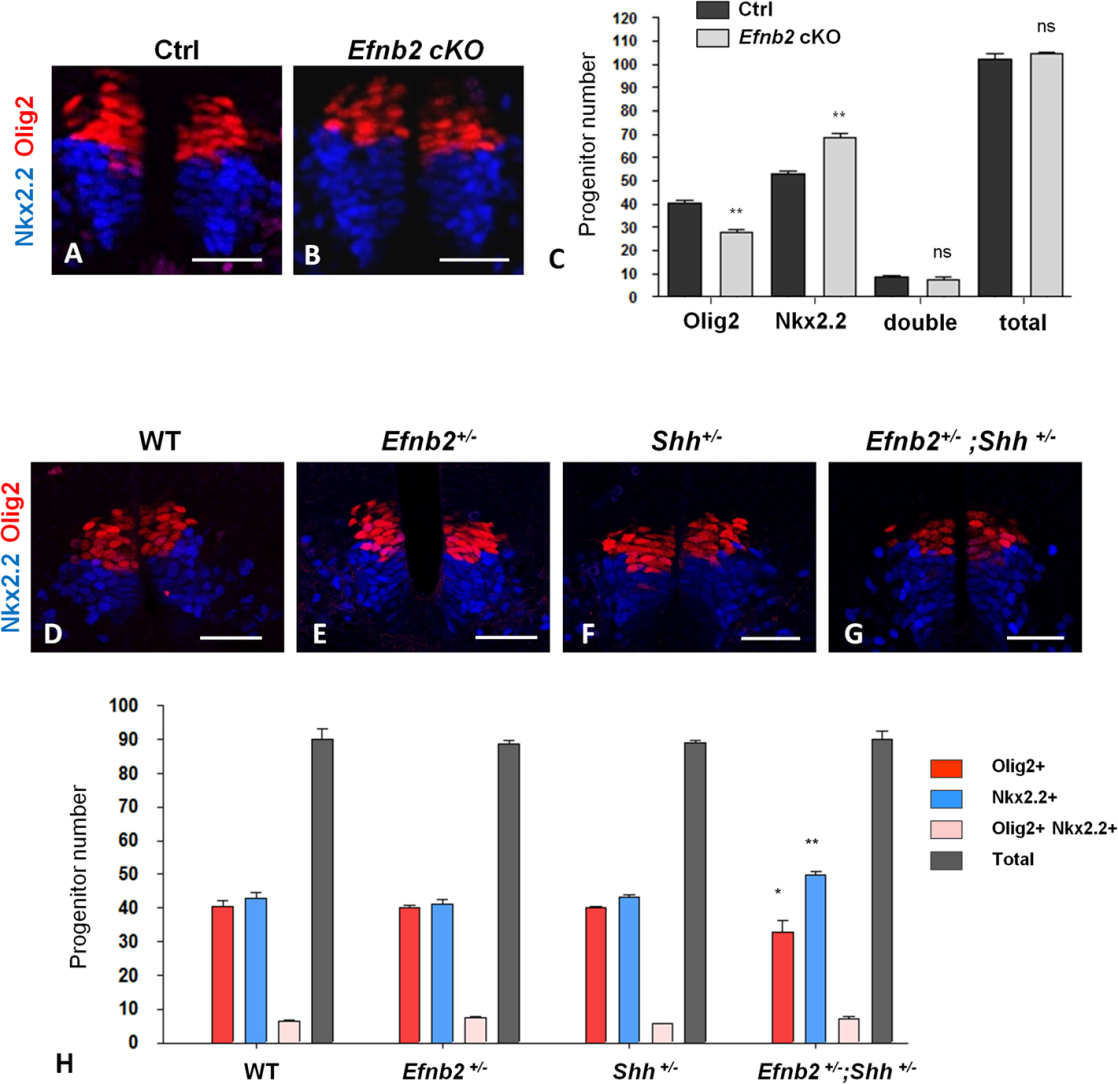
*Efnb2* interacts with *Shh* to control the ratio between pMN and p3 progenitors. **a**, **b**. Transverse sections of *Efnb2* ^*lox/GFP*^ (**a**) and *Efnb2* ^*lox/GFP*^ *;Olig2-Cre* (**b**) E11.5 embryos were immunostained to detect Olig2 (*red*) and Nkx2.2 (blue). **c**. The number of Olig2^+^, Nkx2.2^+^ and Olig2^+^/ Nkx2.2^+^ (double) progenitors was quantified (*n*=5 embryos per genotype). Total refers to the sum of Olig2^+^ and Nkx2.2^+^ progenitors. Error bars indicate s.e.m.; **P<0.01 ns= non significant (Mann-Whitney-Wilcoxon test). **d**-**g**. Transverse sections of E11.5 embryos of different genotypes (as indicated) were immunostained for Olig2 (*red*) and Nkx2.2 (*blue*). **h**. Quantification of the number of Olig2^+^, Nkx2.2^+^ and Olig2^+^/ Nkx2.2^+^ (double) progenitors was quantified for each genotype (*n*=5 embryos per genotype). Total refers to the sum of Olig2^+^ and Nkx2.2^+^ progenitors. Error bars indicate s.e.m.; *P<0.05; **P<0.01; (Mann-Whitney-Wilcoxon test). Scale bars: 50 μm.

An alternate possible cause for the decreased number of Olig2^+^ progenitors at later stages of development could be that a fraction of progenitors wrongly acquire a non-pMN identity after E9.5. To assess this possibility we quantified the number of Nkx2.2^+^ progenitors in E11.5 *Efnb2* cKO embryos and observed that the decreased number of Olig2^+^ progenitors was matched by an increased number of Nkx2.2^+^ progenitors (Fig. 5a-c). Importantly, this was not due to an increase in the number of progenitors of mixed identity (expressing both iTFs) which was similar in control and *Efnb2* cKO embryos (Fig. 5c). Remarkably, the total number of pMN+p3 progenitors was also similar in control and *Efnb2* cKO embryos (Fig. 5a-c), strongly suggesting that in absence of ephrinB2, a fraction of progenitors wrongly acquire the p3 identity (Nkx2.2^+^) at the expense of the pMN identity (Olig2^+^). To challenge this interpretation, we tested for a potential genetic interaction between *Efnb2* and *Shh*, a key player in the specification of p3 and pMN identities [2]. First, we verified that *Shh* expression pattern was not changed in *Efnb2* mutants (Additional file 3: Figure S1). Next, we quantified the number of pMN and p3 progenitors in single *Efnb2* and *Shh* heterozygotes or in compound heterozygote embryos. While the number of Olig2^+^ and Nkx2.2^+^ progenitors in *Efnb2* ^*+/-*^ and *Shh* ^*+/-*^ heterozygous embryos was equivalent to wild type embryos, *Efnb2* ^*+/-*^ ; *Shh* ^*+/-*^ double heterozygous embryos exhibited a phenotype similar to *Efnb2* cKO embryos, with a decrease in Olig2^+^ balanced by an increase in Nkx2.2^+^ progenitors (Fig. 5d-h). These results establish that ephrinB2 and Shh interact genetically to control the ratio between pMN and p3 progenitors in the ventral spinal cord.

### EphrinB3 inversely controls the ratio between pMN and p3 identities

**Fig. 6.**
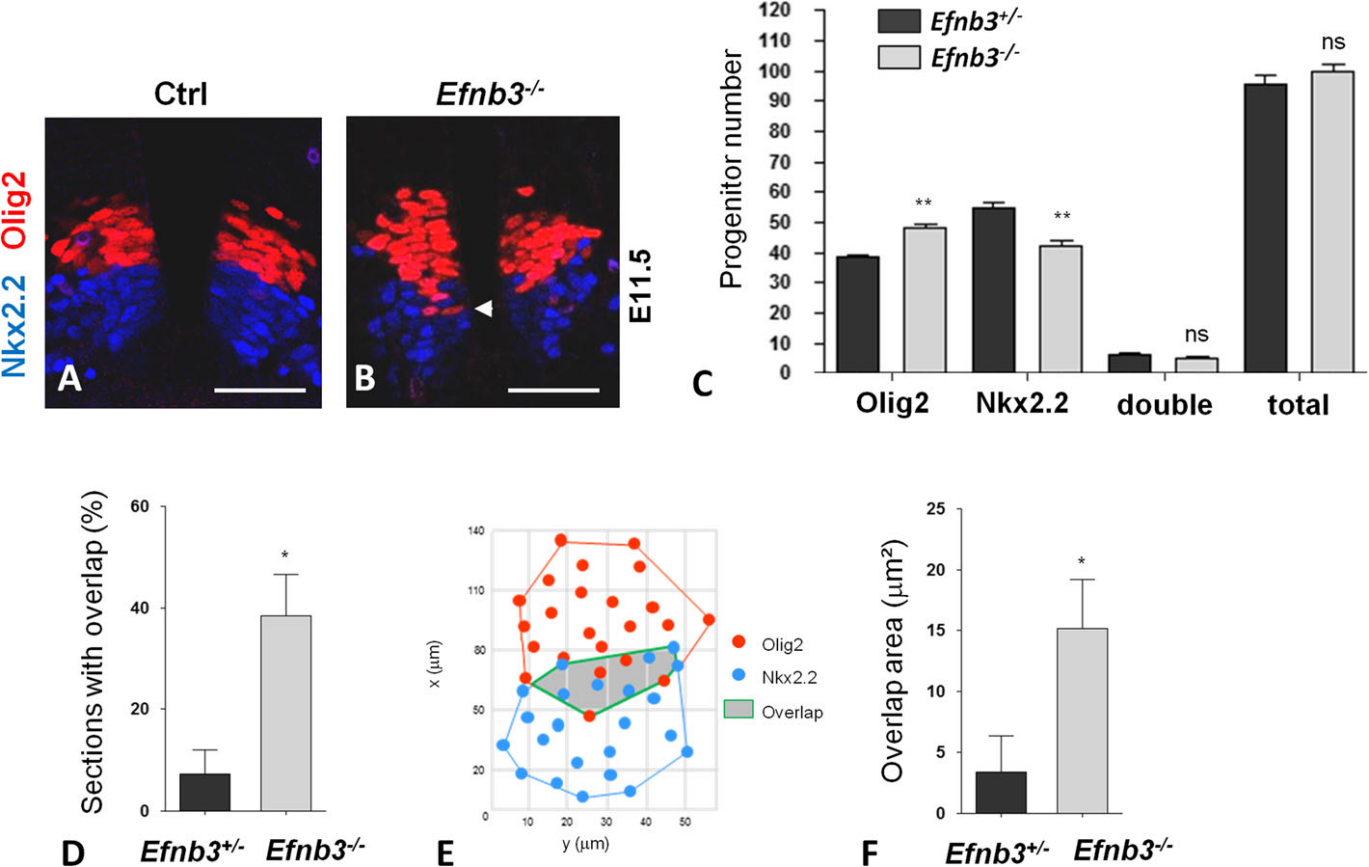
EphrinB3 inversely controls the ratio between pMN and p3 progenitors. **a**, **b**. Transverse sections of *Efnb3* ^*+/-*^ (**a**) and *Efnb3* ^*-/-*^ (**b**) E11.5 embryos were immunostained to detect Olig2 (*red*) and Nkx2.2 (*blue*). **c**. The number of Olig2^+^, Nkx2.2^+^ and Olig2^+^/ Nkx2.2^+^ (double) progenitors was quantified. Total refers to the sum of Olig2^+^ and Nkx2.2^+^ progenitors. **d**. Quantification of the proportion of sections showing an overlap in *Efnb3* ^*+/-*^ and *Efnb3* ^*-/-*^ embryos. **e**. Example of spatial positioning of Olig2^+^and Nkx2.2^+^ progenitors in an overlap situation in an *Efnb3* ^*-/-*^ embryo. **f**. Quantification of the surface of overlap between Olig2^+^ and Nkx2.2^+^ domains in *Efnb3* ^*+/-*^ and *Efnb3* ^*-/-*^ embryos. Error bars indicate s.e.m. (*n*=5 embryos per genotype); *P<0.05; **P<0.01; (Mann-Whitney-Wilcoxon test), ns: non significant. Scale bars: 50 μm.

The above data suggests that expression of ephrinB2 in pMN is required to impose the pMN identity (Olig2^+^). As shown in Fig. 2, ephrinB3 is highly expressed in p3 progenitors (Fig. 2q-s). To test whether ephrinB3 plays a similar role in controlling p3 progenitor identity, we performed immunostaining for Nkx2.2 and Olig2 in *Efnb3* ^*-/-*^ (KO) E11.5 embryos. Quantification of the number of Olig2^+^ and Nkx2.2^+^ progenitors showed that loss of *Efnb3* led to a decrease in the number of Nkx2.2^+^ progenitors which was balanced by an increase in Olig2^+^ progenitors (Fig. 6a-c). Importantly, the number of progenitors with a mixed identity and the total number of p3+pMN progenitors was unchanged in *Efnb3* KO compared to *Efnb3* ^*+/-*^ embryos (Fig. 6c), suggesting that a fraction of progenitors wrongly acquired the pMN at the expense of p3 identity in absence of ephrinB3. No change in progenitor numbers was observed at E9.5 (data not shown) and the increase in pMN number in *Efnb3* KO correlated with an increase in MN numbers (Additional file 3: Figure S2A-D). Interestingly, in addition to a change in the number of pMN and p3 progenitors, intermingling between these progenitors was observed in an increased fraction of sections from *Efnb3* KO compared to sections from *Efnb3* ^*+/-*^ embryos (Fig. 6b, d). To further quantify this phenotype, we measured surfaces encompassing all Olig2^+^ or Nkx2.2^+^ nuclei on multiple transverse sections of *Efnb3* ^*+/-*^ and *Efnb3* KO embryos and deduced their region of overlap (Fig. 6e, f). The surface of overlap between Olig2^+^ and Nkx2.2^+^ domains was increased in *Efnb3* KO compared to control embryos (Fig. 6e, f), confirming intermingling between Olig2^+^ and Nkx2.2^+^ progenitors in absence of ephrinB3. On the contrary, no overlap between Olig2^+^ and Nkx2.2^+^ domains was detected in *Efnb2* cKO (Additional file 3: Figure S3). Altogether, these results indicate that similar to ephrinB2, ephrinB3 is required to control the ratio between p3 and pMN progenitors and that in addition, ephrinB3 is required to maintain a sharp boundary between pMN and p3 progenitor domains.

## Discussion

While early steps of ventral pMN specification have been extensively studied, highlighting the critical role of Shh, mechanisms that control the number of ventral progenitors over time are less well characterized. Here we show that Eph:ephrin signaling is required to precisely control the number of pMN (and p3) progenitors at late stages of development (after E9.5). It has been proposed previously that the modulation of p3 and pMN numbers after E9.5 is driven mainly by differentiation and/or proliferation, which vary over time and according to progenitor types [9]. Despite the fact that a number of studies in the developing and adult cortex have shown a role for Eph:ephrinB signaling in controling the balance between proliferation and differentiation of neural progenitors in the cerebral cortex [18], our data unexpectedly show that changes in pMN progenitor numbers in ephrin mutants are not due to alterations of proliferation or differentiation rates.

**Fig. 7.**
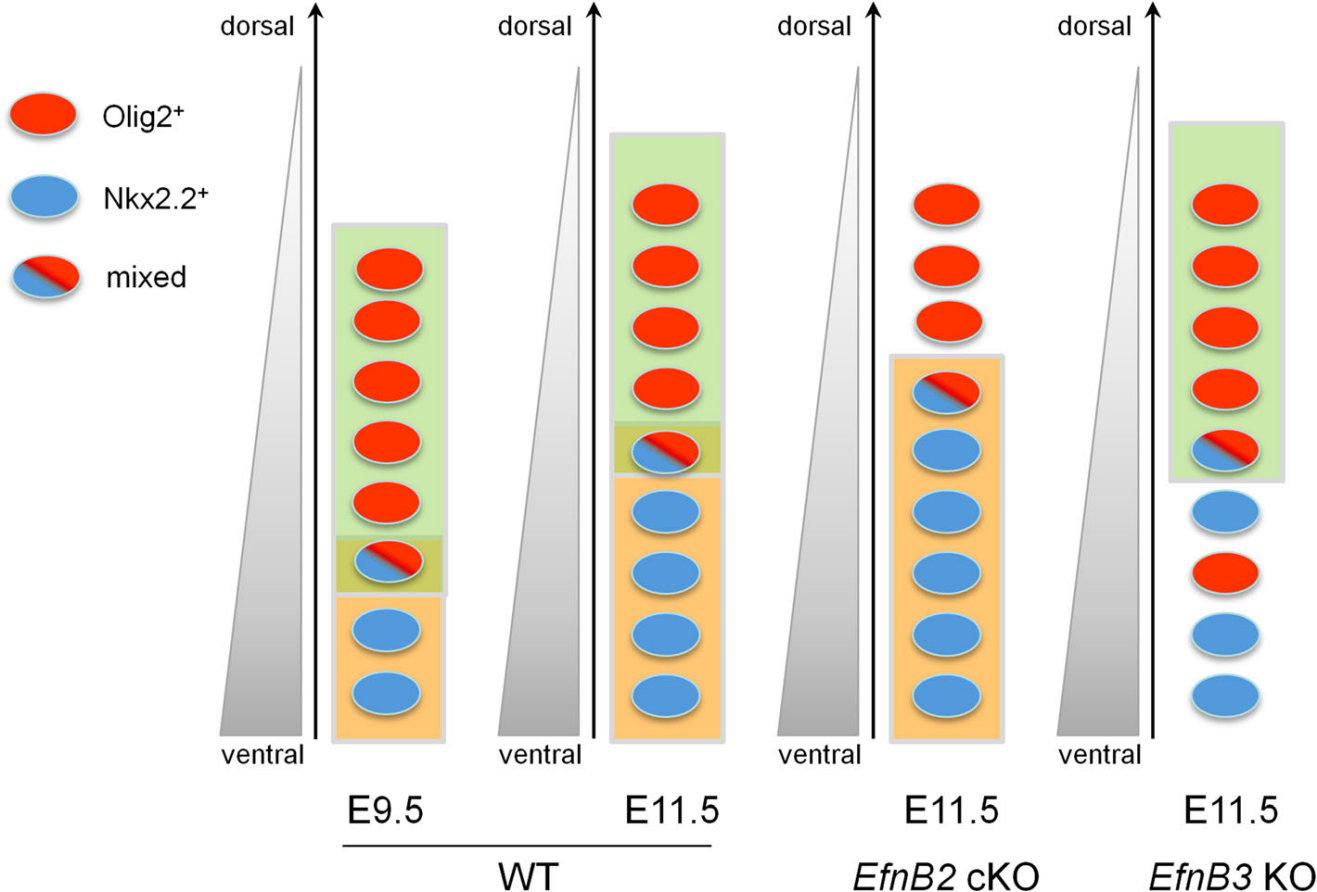
Schematized representation of phenotypes. Schematized representation of transverse sections of the spinal cord showing the dorso-ventral position of p3 (blue), pMN (red) and mixed identity (blue/red) progenitors at different developmental stages and in different genetic backgrounds. Cartoons show 1) the evolution of p3 and pMN numbers over time in wild type embryos and 2) the phenotypes observed in *Efnb2* and *Efnb3* mutants compared to WT at E11.5. Shh gradient (grey) is represented on the left hand side, while domains of ephrinB2 and ephrinB3 expression in progenitors are represented in green and orange, respectively. In *Efnb2* cKO and in *Efnb2;Shh* trans-heterzygotes (not shown), pMN progenitors are fewer while the number of p3 progenitors is increased. Conversely, in *Efnb3* KO, less Nkx2.2^+^ and more Olig2^+^ progenitors are present. In addition, pMN and p3 progenitors are intermingled in *Efnb3* KO.

Instead, our data indicate that ephrinB2 and ephrinB3 are respectively required to impose pMN and p3 identities at later stages of development (Fig. 7). What could be the underlying mechanisms? It has been shown previously that progenitor identity has to be actively maintained after initial specification. Indeed, mouse mutants in which Shh signaling is altered after ventral identities have been assigned exhibit a progressive loss of Olig2^+^ progenitors (and to a lesser extend Nkx2.2^+^ progenitors) indicating that continuous Shh signaling is required to maintain pMN (and p3) identity [4, 7, 8]. Maintenance of identity could thus be one mechanism requiring cooperation between ephrins and Shh. However, we observed that the change in progenitor number in absence of ephrinB2 and ephrinB3 concerns only a small fraction of p3 and pMN progenitors, the majority of which maintain a correct identity in both ephrin mutants. An alternative possibility is thus that after E9.5, a fraction of progenitors requires cooperation between Shh and ephrins to commit to a specific fate. It is interesting to speculate that cells of mixed identity, which represent a small fraction of p3+pMN progenitors at all stages analyzed and are located close to the p3/pMN boundary, could be such a population susceptible to adopt one or the other identity depending on external signals. In cooperation with Shh signaling, EphrinB2 or ephrinB3 may tilt the balance in Olig2 and Nkx2.2 expression and due to the repressive regulatory loop between these iTFs, a shift in expression of one iTF would be amplified and result in commitment to a specific fate. In support to this, lineage tracing studies have shown that the majority of pMN progenitors derive from cells that transiently activate an enhancer for *Nkx2.2* supporting the notion that pMN and p3 progenitors share a common origin [12]. In addition, it has been shown that *Olig2* ^*-/-*^ embryos exhibit only a mild increase in the size of the Nkx2.2^+^ domain [5], suggesting that only a small fraction of progenitors are competent to adopt a p3 fate even in complete absence of Olig2. Further, a similar shift in the ratio between Nkx2.2^+^ and Olig2^+^ progenitors has been described in *Tcf3* ^*-/-*^ *;Tcf4* ^*-/-*^ double mutants and this was linked to the role of Tcf3/4 in inhibiting Nkx2.2 expression in progenitors fated to become pMN [12]. Because Eph:ephrin signaling is a cell-to-cell signaling pathway, this function would be akin to the resolution of binary fates that has been described for Notch signaling in other neural contexts [28]. Of note, this role of ephrinB2 and ephrinB3 in progenitor identities is consistent with their expression patterns in pMN and p3 progenitors, respectively. However, ventral progenitors are in contact with newly generated neurons which also express ephrinB2 and ephrinB3, it is thus possible that the neuronal expression of these ligands may also contribute to maintain a correct ratio between p3 and pMN identities.

As a consequence of these changes in progenitor identities, *Efnb2* and *Efnb3* mutants exhibit opposite alterations in MN numbers at E12.5. It would be interesting to assess whether these changes are still present postnataly, however, postnatal changes in MN number may be due to distinct mechanisms, for instance a decrease in MN number associated with cell death at later stages than those analyzed here has been described in *EphA4* ^*-/-*^ mutants [29].

Traditionally, the role of Eph:ephrin signaling in specification processes has been linked to its function in boundary maintenance. For instance, a recent study has shown that loss of ephrinB2 in the developing cochlea leads to a switch in cell identity from supporting cell to hair cell fate and this was attributed to the mis-positioning of supporting cells into the hair cell layer [30]. No mis-positioning of p3 and pMN progenitors was observed in the *Efnb2* cKO mutants analyzed here. Whether excision of *Efnb2* in all neural tube progenitors would lead to a similar phenotype remains an open question. Here, we observed mis-positioning of progenitors only in ephrinB3 mutants although both ephrinB2 and ephrinB3 mutants exhibited changes in p3 and pMN progenitor ratio, indicating that resolution of identity is independent of mis-positioning. This is consistent with a growing number of published studies reporting a role for Eph:ephrin signaling in lineage commitment or cell fate maintenance via the modulation of intracellular signal transduction pathways and gene expression, independently of cell sorting at boundaries [31–36]. Another possibility, consistent with the genetic interaction between *Efnb2* and *Shh*, could be that ephrins impact on Shh signal transduction cascade as was recently described for Notch [37, 38]. In fact, genetic interaction between Shh and cell surface proteins has been reported previously and such studies identified Gas1, Cdo and Boc as components of the Shh signaling pathway [7, 39, 40]. In this context, it would be interesting to test for a genetic interaction between *Efnb3* and *Shh* in the control of p3 and pMN progenitor identity and positioning.

## Conclusions

In conclusion, our study shows that ephrinB2 and ephrinB3 are required to control progenitor identities in the ventral spinal cord and suggests a role for Eph:ephrin signaling in refining morphogen-dependent tissue patterning.

## Additional files

Additional file 1: Supplemental Code. (PDF 42 kb)

Additional file 2: Table S1. Figure-by-figure details on sample size (DOCX 13 kb)

Additional file 3: Figure S1–3. Expression of *Shh* is not changed in ephrin mutants*. (PDF 330 kb)*

## Abbreviations

cKO: Conditional knock-out
h: Hour
kg: Kilogram
KO: Knock-out
mg: Milligram
ml: Milliliter
MN: Motor neurons
OLP: Oligodendrocyte precursors
Shh: Sonic hedgehog
WT: Wild type
μm: Micrometer
