# Supplementary material for: Eph/Ephrin Signaling Controls Progenitor Identities In The Ventral Spinal Cord
Source: Neural Dev. 2017 Jun 8;12:10. doi: 10.1186/s13064-017-0087-0 (PMC5463316; doi:10.1186/s13064-017-0087-0)
Supplement: Supplementary file 1 — Supplemental Code. (PDF 42 kb) [file 13064_2017_87_MOESM1_ESM.pdf]

Supplemental Experimental Procedures

#Data are in a table formatted as: 4 columns (slide, id, micro1 and micro2)

#Slide = hemi-segment neural tube image (1 z confocal); id = **olig** (for Olig2 positive cell), **nkx** (for Nkx2.2 positive cell) or **merge** (for Olig2 + Nkx2.2 double positive); micro1= x (cell coordinate) and micro2= y.

#Clear all

```
rm(list=ls());
```

#defining current folder

setwd("***folder location***")

#Loading data

data<-read.csv('***file.csv*** ',h=T,sep=';')

#Quick look at the data

```
head(data);
```

Slides list

```
slides<-unique(data$slide);
```

Packages loading

```
library(plyr);

library(PBSmapping);
```

Create 2 data.frames, 'Hulls' containing polygon coordinates and 'allpoly' containing polygon area.

```
Hulls<-data.frame();

allpoly<-data.frame(slide=NA, id=NA, poly=NA, aire=NA);

for (S in slides)

{

        # Subset of data for one slide.

        dataS<-subset(data, slide==S);

        # Polygons for the 3 identities.

        find_hull<-function(Data){Data[chull(Data$micro1,Data$micro2),]};

        hullS<-ddply(dataS,c('id'),find_hull);

        # Ad in data.frame 'Hulls'

        Hulls<-rbind(Hulls,hullS);

## Calculate the Area for each identity (id)

        ID<-unique(hullS$id);

        for (i in ID)

        {

                # Subset for each identity.

                tmp<-subset(hullS, id == i);

                # p test the existence of the polygon (== composed for more than 2 points).
```

```
p<-0;
if (nrow(tmp)>2){p<-1};
# Area calculation or area = 0
Aire<-0;
if (p==1)
{	for (s in 2:(length(tmp$micro1)-1))
	{	A <- abs((tmp$micro1[s]-tmp$micro1[1])*(tmp$micro2[s+1]-tmp$micro2[1])-(tmp$micro1[s+1]-
tmp$micro1[1])*(tmp$micro2[s]-tmp$micro2[1]))/2 ;
	Aire <- Aire+A ;
	}
}
# Ad area in the table 'allpoly'
allpoly<-rbind(allpoly, c(S,as.character(i),p,Aire))
}
# Preparation of data for overlap calculation.
data_nkx<-subset(hullS, id =='nkx');
data_olig<-subset(hullS, id =='olig');
data_merge<-subset(hullS, id =='merge');
r_nkx<-nrow(data_nkx);
r_olig<-nrow(data_olig);
r_merge<-nrow(data_merge);
nkx<-data.frame();
olig<-data.frame();
merge<-data.frame();
# Verification if no data, no overlap is possible
if(r_nkx>0)
{
	nkx<-data.frame(PID=rep(1, r_nkx), POS=1:r_nkx, X= data_nkx$micro1, Y= data_nkx$micro2);
}
if(r_olig>0)
{
	olig<-data.frame(PID=rep(1, r_olig), POS=1:r_olig, X= data_olig$micro1, Y= data_olig$micro2);
}
if(r_merge>0)
{
	merge<-data.frame(PID=rep(1, r_merge), POS=1:r_merge, X= data_merge$micro1, Y= data_merge$micro2);
}
```

```
if (nrow(nkx)>2){poly_nkx<-T}else{poly_nkx<-F};
if (nrow(olig)>2){poly_olig<-T}else{poly_olig<-F};
if (nrow(merge)>2){poly_merge<-T}else{poly_merge<-F};
# Name Overlap polygons
name1<-'nkx_olig';
name2<-'nkx_merge';
name3<-'olig_merge';
poly1<-data.frame();
poly2<-data.frame();
poly3<-data.frame();

# Calculate overlap only if polygon exist
if(poly_nkx==T & poly_olig==T)
{
	# Polygon could exist but not cross, use try to force the test.
	poly1<-try(joinPolys(nkx, olig));
	# If error in try there is no overlap.
	if(length(poly1)==0){n<-0}else{n<-nrow(poly1)};
	if (n>0)
	{
		dat1<-data.frame(slide=rep(S,n),id=rep(name1,n),micro1=poly1$X,micro2=poly1$Y);
		# Add corordinates in 'Hulls'.
		Hulls<-rbind(Hulls,dat1);
		p<-1;
		Aire<-0;
		for (s in 2:(length(poly1$X)-1))
		{
			A <- abs((poly1$X[s]-poly1$X[1])*(poly1$Y[s+1]-poly1$Y[1])-(poly1$X[s+1]-poly1$X[1])*(poly1$Y[s]-
poly1$Y[1]))/2;
			Aire <- Aire+A;
		}
	}else{p<-0; Aire<-0}
		# Add area in 'allpoly'.
		allpoly<-rbind(allpoly,c(S,name1,p,Aire));
}else{allpoly<-rbind(allpoly,c(S,name1,0,NULL))};
```

```
        # same for the others polygons overlaps.
        if(poly_nkx==T & poly_merge==T)
        {
                poly2<-try(joinPolys(nkx, merge));
                if(length(poly2)==0){n<-0}else{n<-nrow(poly2)};
                if(n>0)
                {
                        dat2<-data.frame(slide=rep(S,n),id=rep(name2,n),micro1= poly2$X,micro2= poly2$Y);
                        Hulls<-rbind(Hulls,dat2);
                        p<-1;
                        Aire<-0;
                        for (s in 2:(length(poly2$X)-1))
                        {
                                A <- abs((poly2$X[s]-poly2$X[1])*(poly2$Y[s+1]-poly2$Y[1])-(poly2$X[s+1]-poly2$X[1])*(poly2$Y[s]-
poly2$Y[1]))/2;
                                Aire <- Aire+A;
                        }
                }else{p<-0;Aire<-0};
                allpoly<-rbind(allpoly,c(S,name2,p,Aire));
        }else{allpoly<-rbind(allpoly,c(S,name2,0,0))};

        if(poly_olig==T & poly_merge==T)
        {
                poly3<-try(joinPolys(olig, merge));
                if(length(poly3)==0){n<-0}else{n<-nrow(poly3)};
                if (n>0)
                {
                        dat3<-data.frame(slide=rep(S,n),id=rep(name3,n),micro1= poly3$X,micro2= poly3$Y);
                        Hulls<-rbind(Hulls,dat3);
                        p<-1;
                        Aire<-0;
                        for (s in 2:(length(poly3$X)-1))
                        {
                                A <- abs((poly3$X[s]-poly3$X[1])*(poly3$Y[s+1]-poly3$Y[1])-(poly3$X[s+1]-poly3$X[1])*(poly3$Y[s]-
poly3$Y[1]))/2;
                                Aire <- Aire+A;
                        }
```

```
				}else{p<-0;Aire<-0}
				allpoly<-rbind(allpoly,c(S,name3,p,Aire));
		}else{allpoly<-rbind(allpoly,c(S,name3,0,0))};
}
# Errors are possible but ‘Try’ is here for that.
# Erase and save tables on .rda
Hulls<-na.omit(Hulls);
allpoly<-na.omit(allpoly);

save(Hulls, file='01-Hulls.rda');
save(allpoly, file='01-Allpoly.rda');

write.csv(Hulls, file='01-Hulls.csv');
write.csv(allpoly, file='01-Allpoly.csv');
```
