# Supplementary material for: Eph/Ephrin Signaling Controls Progenitor Identities In The Ventral Spinal Cord
Source: Neural Dev. 2017 Jun 8;12:10. doi: 10.1186/s13064-017-0087-0 (PMC5463316; doi:10.1186/s13064-017-0087-0)
Supplement: Supplementary file 2 — Figure-by-figure details on sample size (DOCX 13 kb) [file 13064_2017_87_MOESM2_ESM.docx]

**Supplemental Table 1. Figure-by-figure details on sample size.**

| **Figure 3G** | nb Z-sections | 242 |
| --- | --- | --- |
|  | nb tissue sections | 16-18 |
|  | nb embryos E9.5 | 3 |
| **Figure 3G** | nb Z-sections | 255 |
|  | nb tissue sections | 23-28 |
|  | nb embryos E11.5 | 5 |
| **Figure 3J** | nb Z-sections | 570 |
|  | nb tissue sections | 55-59 |
|  | nb embryos | 6 |
| **Figure 3K** | nb Z-sections | 570 |
|  | nb tissue sections | 55-59 |
|  | nb embryos | 6 |
| **Figure 4C** | nb Z-sections | 240 |
|  | nb tissue sections | 20 |
|  | nb embryos | 4 |
| **Figure 4F** | nb Z-sections | 258 |
|  | nb tissue sections | 20-21 |
|  | nb embryos | 4 |
| **Figure 4G** | nb Z-sections | 258 |
|  | nb tissue sections | 20-21 |
|  | nb embryos | 4 |
| **Figure 5C** | nb Z-sections | 255 |
|  | nb tissue sections | 23-28 |
|  | nb embryos | 5 |
| **Figure 6C** | nb Z-sections | 511 |
|  | nb tissue sections | 30-32 |
|  | nb embryos | 5 |
| **Figure 6D** | nb Z-sections | 181 |
|  | nb tissue sections | 25 |
|  | nb embryos | 5 |
| **Sup Figure 2C** | nb Z-sections | 735 |
|  | nb tissue sections | 72-75 |
|  | nb embryos | 8 |
| **Sup Figure 2D** | nb Z-sections | 735 |
|  | nb tissue sections | 72-75 |
|  | nb embryos | 8 |
| **Sup Figure 3A** | nb Z-sections | 180 |
|  | nb tissue sections | 25 |
|  | nb embryos | 5 |
| **Sup Figure 3B** | nb Z-sections | 180 |
|  | nb tissue sections | 25 |
|  | nb embryos | 5 |
|  | nb embryos | 4 |
