# Supplementary material for: Eph/Ephrin Signaling Controls Progenitor Identities In The Ventral Spinal Cord
Source: Neural Dev. 2017 Jun 8;12:10. doi: 10.1186/s13064-017-0087-0 (PMC5463316; doi:10.1186/s13064-017-0087-0)
Supplement: Supplementary file 3 — Expression of Shh is not changed in ephrin mutants. (PDF 330 kb) [file 13064_2017_87_MOESM3_ESM.pdf]

Sup Figure 1, Laussu et al.

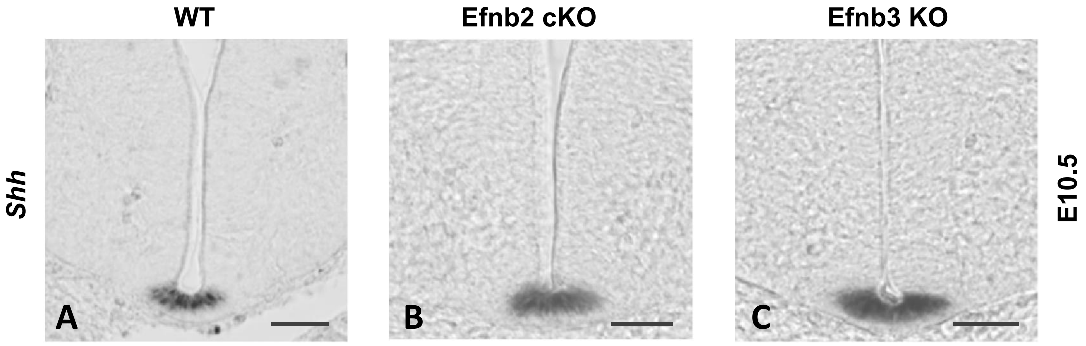

Sup Figure 1. Expression of *Shh* is not changed in ephrin mutants.
Expression of *Shh* was analyzed by in situ hybridization in E10.5 wild type, *Efnb2* cKO and *Efnb3* KO as indicated. No change in *Shh* expression pattern was detected in ephrin mutants embryos. Scale bar: 50 µm.

Sup Figure 2, Laussu et al.

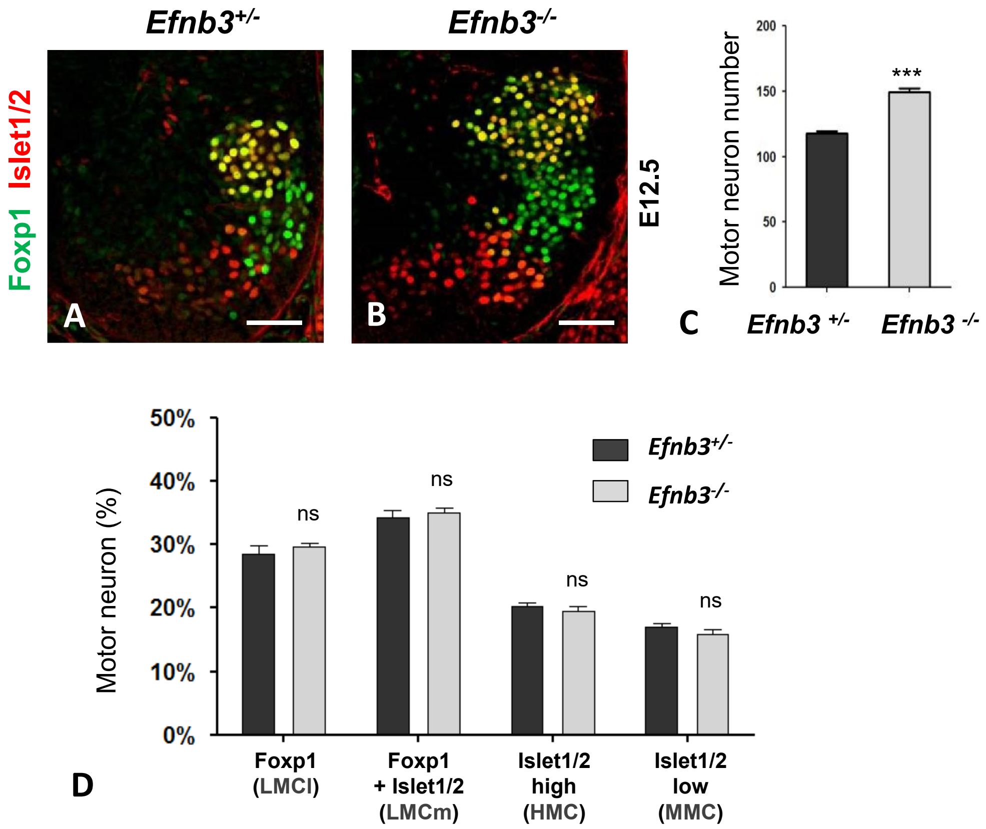

Sup Figure 2. Increased number of MN in *Efnb3* KO.

A, B. Transverse sections of E12.5 *Efnb3*$^{+/-}$ (A) and *Efnb3*$^{-/-}$ (B) embryos were immunostained to detect Foxp1 (green) and Islet 1/2 (red). C. Quantification of the total number of motor neurons (Foxp1^{+} and Islet 1/2^{+}) in both genotypes. D. Repartition of motor neurons in motor columns in both genotypes. Error bars indicate s.e.m. (n=6 embryos per group); ***P<0.001; ns= non significant (Mann-Whitney-Wilcoxon test).

Sup Figure 3, Laussu et al.

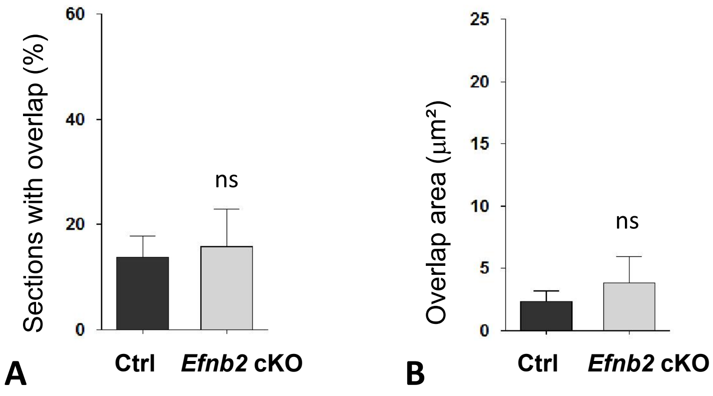

Sup Figure 3. No intermingling between pMN and p3 progenitors in *Efnb2* cKO.
The following quantifications were performed on immunostainings shown in Figure 5. A. Quantification of the proportion of sections showing an overlap in control and *Efnb2* cKO embryos. B. Quantification of the surface of overlap between $Olig2^{+}$ and $Nkx2.2^{+}$ domains in control and *Efnb2* cKO embryos. Error bars indicate s.e.m. (n=5 embryos per genotype); ns: non significant (Mann-Whitney-Wilcoxon test).
